# Effect of chitosan on *Toxoplasma gondii* infection: A systematic review

**DOI:** 10.1016/j.parepi.2020.e00189

**Published:** 2020-10-21

**Authors:** Kourosh Cheraghipour, Leila Masoori, Fatemeh Ezzatkhah, Iraj Salimikia, Sana Amiri, Ali Safar Makenali, Farshad Taherpour, Hossein Mahmoudvand

**Affiliations:** a Razi Herbal Medicines Research Center, Lorestan University of Medical Sciences, Khorramabad, Iran; b Department of Laboratory Sciences, School of Allied Medicine, Lorestan University of Medical Sciences, Khorramabad, Iran; c Department of Laboratory Sciences, Sirjan School of Medical Sciences, Sirjan, Iran; d Veterinary Epidemiologist, Chief Veterinary Officer, Tehran, Iran; e Hepatitis Research Center, Lorestan University of Medical Sciences, Khorramabad, Iran

**Keywords:** Nanoparticles, Toxoplasmosis, Treatment, *In vitro*, *In vivo*, Natural products

## Abstract

**Background:**

The preferred treatment for management of toxoplasmosis is the combined use of pyrimethamine and sulfadiazine. However, there are a wide number of adverse side effects with these medications. Recent research has focused on the use of chitosan for the treatment of *Toxoplasma gondii* infections. This review was performed to obtain a better understanding of the *in vivo* and *in vitro* effects of chitosan on *T. gondii* strains.

**Methods:**

The current study was carried out according to the PRISMA guideline and registered in the CAMARADES-NC3Rs Preclinical Systematic Review and Meta-analysis Facility (SyRF) database. The search was performed in five scientific databases, including Scopus, PubMed, Web of Science, EMBASE, and Google Scholar, with date limits of 1992 to December 2019. The search was restricted to articles published in the English language. The words and terms searched were “*Toxoplasma gondii*”, “Chitosan”, “nanoparticles” and “anti-toxoplasmosis” with AND or OR.

**Results:**

Of 2500 manuscripts, 9 met the eligibility criteria for review. All studies used the RH strain of *T. gondii*, with Me49 and PRU each included in one study. Five studies (56%) were performed *in vivo*, one study *in vitro* and 3 studies included *in vivo* and *in vitro* tests.

**Conclusion:**

Considering the low toxicity and the high inhibitory potency of chitosan against *T. gondii*, chitosan nanoparticles show potential as an alternative treatment for *T. gondii infections*.

## 1 Introduction

*Toxoplasma gondii* is a well-known, protozoan that infects up to one third of the human population in all countries of the world (Dubey, 2004). The primary modes of transmission to humans are: (i) consumption of raw or undercooked meat contaminated with tissue cysts; (ii) ingestion of sporulated oocysts with food; or (iii) congenitally, from mother to fetus during pregnancy (Hill and Dubey, 2002; Khryanin et al., 2015). While most infections are asymptomatic, some can result in severe clinical issues and, in the case of congenital infections, even death (Lewis et al., 2015; Mahmoudvand et al., 2015). The current recommended treatment for management of toxoplasmosis is the combined use of pyrimethamine and sulfadiazine; however, these medications can result in adverse side effects such as osteoporosis, liver and kidney complications and also is contraindicated in early pregnancy (Mahmoudvand et al., 2016a; Mahmoudvand et al., 2016c; Antczak et al., 2016). Alternative treatments such as spiramycin, and antibiotics like fluoroquinolones were included.

Chitosan is a group of partially and completely deacetylated chitin components which have a wide range of pharmacological properties alone or along with other natural polymers (starch, gelatin, alginates) in the modern medicine, food, agriculture, and cosmetics industries (Montazeri et al., 2017; Wang et al., 2011).

Recently, there has been more research on antimicrobial activity of chitosan (Hosseinnejad and Jafari, 2016). Antimicrobial effects of chitosan have been demonstrated against a broad spectrum of bacteria, filamentous fungi and yeasts, pathogenic viruses, protozoan and helminthic parasites, including *Leishmania* spp., *Cryptosporidium* spp., and *Echinococcus granulosus* metacestodes (Jeon et al., 2001; Guan et al., 2019; Rozman et al., 2019; Rabea et al., 2003; Varshosaz et al., 2018; Mammeri et al., 2018; Torabi et al., 2018). Here, this study was designed to review the effect of chitosan on *T. gondii in vivo* and *in vitro*.

## 2 Materials and methods

### 2.1 Search strategy

The current study was carried out according to the PRISMA guideline and registered in the CAMARADES-NC3Rs Preclinical Systematic Review and Meta-analysis Facility (SyRF) database (Teimouri et al., 2018). The search was performed in five scientific databases, including Scopus, PubMed, Web of Science, EMBASE, and Google Scholar. The search was limited to articles in English published between 1992 and December 2019. The words and terms searched, in combination, were “*Toxoplasma gondii*”, “Chitosan”, “nanoparticles” and “anti-*Toxoplasma*” with AND or OR.

### 2.2 Inclusion criteria and data extraction

**Fig. 1:**
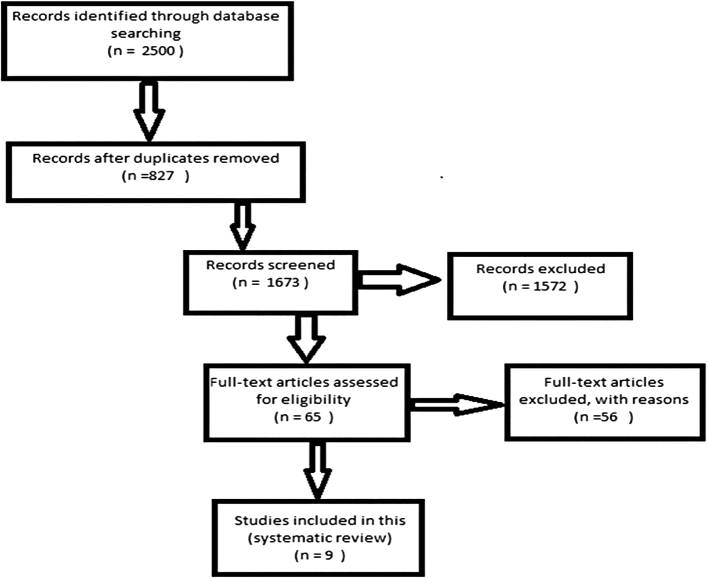
Flowchart describing the study design process.

After removing duplicate publications, three authors independently reviewed the titles and abstracts of the studies, and the approved studies were selected for further analysis. With scientific documentation and at this stage, studies with inadequate information, with lack of evidence, noncompliance with the methods and misinterpretation of the results were excluded from the present study. A flowchart depicts the study design process (Fig. 1). Moreover, the studies with inadequate information, only abstract, failure to match methods with results, and the incorrect interpretation of the results was excluded from the current study. Once an article was identified to be included, the following data were extracted: nanoparticles structure, chitosan formulation, dosage consumption, experimental method and efficacy results.

## 3 Results

Nine papers (1 *in vitro*, 5 *in vivo*, 3 *in vitro* and *in vivo*) from 2003 to 2019, met the inclusion criteria for discussion in this systematic review with the data extracted are presented in Table 1, Table 2. All of the *in vitro* studies were performed with the RH strain while two of the *in vivo* studies included type-2 strains (Me49 and PRU).

**Table 1:** A list of *in vitro* efficacy of chitosan and some its formulations against *T. gondii.*

Nanoparticles structure	Chitosan formulations	Strain	Concentration	Exposure time	Positive control	*In vitro* results	Ref
Chitosan	NM^a^	RH^c^	500,1000 and 2000 ppm	30,60,120 an 180 min	NM	LMW d of chitosan had the most mortality against tachyzoite of RH strain	(Teimouri et al., 2018)
Chitosan	Alginate chitosan calcium phosphate nanocapsules (AEC-CCo-CP-NCs)	RH	10, 20, and 40 μg/mL	24 h	Sulphadiazine, 20 μg/mL	*In vitro* analysis has shown a increase in nitric oxide production and low parasitemia in *in vitro* cell culture model	(Anand et al., 2015)
Chitosan microspheres	Multiple antigenic peptide (MAP) of GRA10^b^ (G10E)	RH	125, 250, and 500 μg/mL)	48 and 72 h	NM	- G10E-CS^e^ increased co-stimulatory molecules in dendritic cells - G10E-CS-increased percent of CD8+/CD4+ - G10E-CS increased levels of IFN-γ^f^ and IL-2^g^	(Sun et al., 2018)
Chitosan	Chitosan-zinc oxide (ZnO) nanoparticles	RH	(25, 50 and 100 U mL^−1^	10 min	NM	CH-ZnO-NPs nanoplatform are efficient analytical tool in the clinical diagnosis and monitoring of toxoplasmosis.	(Medawar-Aguilar et al., 2019)

a Not mentioned.

b Dense granular protein GRA10.

c Standard strain of *Toxoplasma* (type I).

d Low molecular weights.

e Cesium standard.

f Interferon gamma.

g Interleukin 12.

**Table 2:** A list of *in vivo* efficacy of chitosan and some its formulations against *T. gondii.*

Nanoparticles structure	Chitosan formulations	Strain	Concentration	Exposure time	Positive control/dose	Model	Results	Ref
Chitosan	NM^a^	RH	0.2 mL LMW^c^ 0.2 ml MMW2^d^ 0.2 ml HMW3^e^	5 days	Sulfadiazine 400 mg/L/day	BALB/c mice	Growth inhibition rates of tachyzoites in mice receiving LMW, MMW and HMW CS NPs were found to be 86, 84 and 79%, respectively	(Teimouri et al., 2018)
Chitosan	Alginate chitosan calcium phosphate nanocapsules (AEC-CCo-CP-NCs)	RH	1.2% (w/w)	5,10 and 15 days	Sulfadiazine 40 mg/kg/day	BALB/c mice	AEC-CCo-CP-NCs deceared the parasite load in various organs and helped survival of mice till day 25 postinfection.	(Anand et al., 2015)
Chitosan microspheres	Multiple antigenic peptide (MAP) of GRA10^b^ (G10E)	RH Prugniaud strain	667 μg CS^f^ Microsphere and 100 μg of G10E	14 days	NM	BALB/c mice	Increase survival time in group immunized with G10E-CS Immunization with G10E-CS caused protection with prolonged survival in mice model of acute toxoplasmosis and decreases in cyst burden in murine chronic toxoplasmosis.	(Sun et al., 2018)
Chitosan	Spiramycin	RH	400 mg/kg/day 100 mg/kg/day	7 days	Spiramycin	Swiss albino mice	Spiramycin-loaded NPs showed the highest reduction of tachyzoites (about 90% reduction)	(Hagras et al., 2019)
Chitosan	GRA-1 protein GRA1 encoding pDNA	RH	50 μɡ GRA1 pDNA 50 μɡ recombinant GRA1 protein and 100 μɡ chitosan	28 days	NM	C3H/ mice	Oral delivery of vaccines using chitosan as a carrier material appears to be beneficial for inducing an immune response against *T. gondii*	(Bivas-Benita et al., 2003)
Chitosan	Chitosan nanospheres encapsulated with *Toxoplasma* lysate	RH Me49	NM	14 days	NM	Swiss abino mice	- Increase survival time - Increase IgG^g^ and IFN- γ^h^ - Reduction of pathological changes	(El Temsahy et al., 2016)
Chitosan	Chitosan combined with silver (Ag)	RH	100 μg/mL and 200 μg/mL	4 days	Pyrimethamine 0.25 mg/mouse	Swiss albino mice	- Decrease mean numbers of tachyzoites in liver mice immunized with CS NPs - Increase concentration of INF-γ in mice immunized with CS NPs a dose of 200 μg/ml also mice immunized with CS and Ag NPs	(Gaafar et al., 2014)
Chitosan	Spiramycin	RH	NM	7 days	NM	Swiss albino mice	- Spiramycin combined with chitosan nanoparticles has good results compared to single therapy - Increased IgM^i^- TNF-α^j^ and INF-γ	(Hamad et al., 2018)

a Not mentioned.

b Dense granular protein GRA10.

c Low molecular weight.

d Medium molecular weights.

e High molecular weights.

f Cesium standard.

g Immunoglobulin G.

h Interferon gamma.

i Immunoglobulin M.

j Tumor necrosis factor-α.

## 4 Discussion

Currently, toxoplasmosis treatment is often based on a combination of sulfadiazine and pyrimethamine. The finite efficacy of these drugs and their advers side effects highlights the need for novel therapeutic strategies to increase drug efficiency and decrease toxicity of agents (Teimouri et al., 2018; Shubar et al., 2011). In novel medicine, nearly 25% of synthetic drugs are produced using traditional and natural herbs for the treatment of various diseases (Kean and Thanou, 2010; Cowan, 1999). Chitosan is one of the newest synthesized compounds of chitin, acting as a potent antimicrobial agent as well as the presence of natural and non-toxic compounds in various forms (Shubar et al., 2011; Verlee et al., 2017). In some countries, chitosan is used as an edible matter, it has also been approved by the FDA for use as a wound bandage (Torabi et al., 2018; Kean and Thanou, 2010).

In 2016, the effects of chitosan on diminishing the pathogenicity of some parasites such as *Plasmodium berghei*, *Trichomonas gallinae,* and *Giardia* have been studied (Wang et al., 2011; Yarahmadi et al., 2016).

Few of the studies used the same CS formulation, dose, exposure or model, therefore making comparisons challenging. However, these variations demonstrated a variety of activity and the flexibility in the formulations feasible with chitosan nanoparticles.

Low molecular weight nanoparticles at concentrations of 500 and 1000 ppm with 180 min exposure killed all tachyzoites. In addition, treatment of tachyzoites with chitosan nanoparticles *in vitro* and then injecting these tachyzoites to mice increased their span of life for up to two months. Also, higher concentrations (2000 ppm) resulted in morphological changes of the tachyzoites. Also, altered induction of cellular apoptosis in the tachyzoite surface and their structure degradation (Teimouri et al., 2018). As well as, alginate chitosan calcium phosphate bovine lactoferrin nanocapsules (AEC-CCo-CP-bLf-NCs) have been evaluated against *T. gondii.* According to this study, macrophage cell lines treated with 20 μg/mL of apo-blf nanoparticles and sulfadiazine had the highest percentage of nitric oxide production. On the other hand, it decreased the number of intracellular tachyzoites significantly. Evaluation of the different groups treated with AEC-CCo-CP-bLf-NC by quantitative real-time method indicated a reduction of tachyzoites burden compared to the control group. Also, the survival time of mice treated by AEC-CCo-CP-bLf-N continued until day 25 that was statistically significant in comparison to the control group (Anand et al., 2015).

Spiramycin- loaded chitosan nanoparticles were studied for the treatment of experimental acute toxoplasmosis in mice infected with *RH T. gondii*, In this study, a group of mice treated with Spiramycin- loaded chitosan nanoparticles at a dose of 400 mg/mL for 7 days. They had the highest survival time up to 18 days after treatment with nanoparticles. Furthurmore there were no any mortality until 8th day. On the other hand, the rate of peritoneal tachyzoite decrease was 120,130 ± 122,474 which showed a significant decrease in the number of tachyzoites compared to the untreated group (Hagras et al., 2019). In addition, GRA-1 protein and GRA-1 pDNA synthesized from *T. gondii* and loaded with chitosan nanoparticles were tested as vaccines for anti-*Toxoplasma* antibody assay. Anti-GRA-1 antibodies including IgG2a/IgG1 that chitosan prevented enzymatic degradation of GRA-1 protein as well as DNA and in turn increased anti-toxoplasmosis immune response (Bivas-Benita et al., 2003). Group vaccination of mice using Crude *Toxoplasma* lysate vaccine (CTLV) encapsulated with chitosan nanospheres or with complete Freund's adjuvant accomplished. And the end challenge of mice with RH and Me49 strains increased the survival time of these groups for 80 and 57 days, respectively. The life span of these vaccinated groups was significantly increased compared to the control group. Also, the level of INF-γ was significantly increased in the CTLV-encapsulated groups with chitosan and adjuvant Freund compared to the control group and in the vaccinated group. With CTLV encapsulated chitosan nanospheres (CTLVECNS) and then challenged with Me49 strain, the number of smaller cysts formed by traverse inflammatory cells in the brain of this group was not seen (El Temsahy et al., 2016).

Effectiveness of nanoparticles against *T. gondii* infection has been shown to influence and reduce tachyzoite parasitic load *in vitro* environments, as in the study of Gaafar et al. (2014). organs including liver indicated that treatment with chitosan nanoparticles at a concentration of 200 μg/ml reduced the mean number of tachyzoites by 6.36 ± 0.369.

The mean number of tachyzoites in the pretreatment group was 7.2 ± 1.0834. Chitosan nanoparticles also significantly increased the level of INF-γ cytokine in the chitosan treated group with the concentration of 200 μg/ml as well as chitosan nanoparticles treated with silver compound compared to the control group (Gaafar et al., 2014).

In addition, the use of nanoparticles named chitosan Microsphere, in which the Multiple antigenic peptides were synthesized from GRA10, played an important role in the immunization of mice treated with (G10E-CS). This study indicated that BALB/c mice injected intramuscularly (G10E-CS) with RH and PRU strains for two weeks and after these injections, were treated with survival time in the RH challenge group for 21 days. That this significantly higher than the other groups.

On the other hand, the G10E-CS treated mice group which were then challenged with PRU strain had the lowest brain cyst rate of 882±194 compared to the other treated groups .G10E-CS also increased dendritic cells *in vitro* and elevated INF-γ cytokines. It also decreased IL-4 and IL-10 cytokines. The results of this study could be lead to the effectiveness of chitosan microspheres as an effective system for delivering antigenic peptides to dendritic cells in research and development of new vaccines preventing *T. gondii* infections (Sun et al., 2018).

Studies using spiramycin-loaded chitosan nanoparticles on mice treated with RH for 7 days after infection showed an increase in cytokines such as INF-γ, (TNF-α), and an increase in IgM against parasitoid tachyzoites. Results implied improvement and enhancement of the immune system in mice treated with spiramycin-loaded chitosan nanoparticles (Hamad et al., 2018).

Chitosan nanoparticles have been studied as a natural polymer for the stabilization of metallic nanoparticles. Chitosan-coated zinc oxide by binding to IgG antibodies can evaluate the low level of this antibody using a microfluidic LIF immunosensor device. This method develops chitosan-coated zinc oxide nanoparticles as an efficient and effective analytical tool in the detection and monitoring of toxoplasmosis clinical (Medawar-Aguilar et al., 2019). There are some limitaions for this study including (i) the effects of chitosan nanoparticles should be evaluated more widely in animal clinical trial; (ii) the loading capacity of chitosan nanoparticles to protect compounds such as polypeptides and other substances synthesized as an anti-toxoplasmosis vaccine against degradation enzymes such as DNase I should be increased; (iii) more extensive studies should be conducted on the use of chitosan as an effective drug for acute and chronic experimental models of *T. gondii*.

## 5 Conclusion

Considering the low toxicity and the high inhibitory potency of chitosan against *T. gondii,* chitosan nanoparticles show potential as an alternative treatment for *T. gondii* infections. Chitosan nanoparticles in combination with other therapies also could provide a higher level of efficacy. However, extensive *in vivo* and clinical trials on human subjects are needed to approve the impact of chitosan nanoparticles for treatment of toxoplasmosis.

## Funding

None.

## Declaration of Competing Interest

The authors declare no conflict of interest.
